# Portable fixed dynamometry: towards remote muscle strength measurements in patients with motor neuron disease

**DOI:** 10.1007/s00415-020-10366-9

**Published:** 2020-12-23

**Authors:** Jaap N. E. Bakers, Leonard H. van den Berg, Toju G. Ajeks, Maxine J. Holleman, Jill Verhoeven, Anita Beelen, Johanna M. A. Visser-Meily, Ruben P. A. van Eijk

**Affiliations:** 1 Department of Neurology, UMC Utrecht Brain Centre, University Medical Centre Utrecht, Heidelberglaan 100, 3584 CX Utrecht, The Netherlands; 2 Centre of Excellence for Rehabilitation Medicine, UMC Utrecht Brain Centre, University Medical Centre Utrecht, and De Hoogstraat Rehabilitation, Utrecht, The Netherlands; 3 Department of Rehabilitation, Physical Therapy Science and Sports, UMC Utrecht Brain Centre, University Medical Centre Utrecht, Utrecht, The Netherlands; 4 Biostatistics and Research Support, Julius Centre for Health Sciences and Primary Care, University Medical Centre Utrecht, Utrecht, The Netherlands

**Keywords:** Fixed dynamometry, Motor neuron disease, Muscle strength, Remote monitoring, Reliability study

## Abstract

**Background:**

We aimed to determine (1) the test–retest reliability of a newly developed portable fixed dynamometer (PFD) as compared to the hand-held dynamometer (HHD) in patients with motor neuron disease (MND) and (2) the PFD’s ability to reduce possible examiner-induced ceiling effects.

**Methods:**

Test–retest reliability of isometric muscle strength of the quadriceps was measured in patients with MND and non-neurological controls using the HHD and PFD. Reliability was estimated by the intraclass correlation coefficient (ICC) and standard error of measurement (SEM) using linear mixed effects models, and the Bland–Altman method of agreement.

**Results:**

In total, 45 patients with MND and 43 healthy controls were enrolled in this study. The ICC of the PFD was excellent and similar in both patients and controls (ICC _Patients_ 99.5% *vs.* ICC _Controls_ 98.6%) with a SEM of 6.2%. A strong examiner-induced ceiling effect in HHD was found when the participant’s strength exceeded that of examiner. Employing the PFD increased the range of muscle strength measurements across individuals nearly twofold from 414 to 783 N.

**Conclusions:**

Portable fixed dynamometry may significantly reduce examiner-induced ceiling effects, optimize the standardization of muscle strength testing, and maximize reliability. Ultimately, PFD may improve the delivery of care due to its potential for unsupervised, home-based assessments and reduce the burden to the patient of participating in clinical trials for MND or other neuromuscular diseases.

## Introduction

Progressive muscle weakness is the hallmark of motor neuron disease (MND) [1]. Muscle strength testing has, therefore, a central role in monitoring MND progression [2–4]. Isometric muscle strength testing using the Hand-Held Dynamometer (HHD) is preferred to the Medical Research Council (MRC) scale due to its increased objectivity and sensitivity [3, 5–7]. Despite its user-friendliness, portability and cost-effectiveness, the HHD’s reliability depends on the technique and strength of the examiner [8–13]. These limitations become especially apparent in strong muscle groups such as the quadriceps, resulting in possible ceiling effects and a reduced sensitivity for quantifying MND progression in early disease stages [14, 15].

Reducing examiner variability may, therefore, significantly reduce measurement error and optimize the sensitivity of muscle strength testing in MND. Fixed dynamometry (i.e., fixation of the dynamometer in a rigid structure) has been shown to alleviate the limitations of the HHD, but currently available systems still require a trained examiner and hospital visits [16–24].

Given the current transition to home-based assessments (i.e., remote monitoring) [25, 26], and the accompanying need for reliable and unsupervised measurements of disease progression [27, 28], we developed a portable fixed dynamometer (PFD). The PFD was developed to evaluate quadriceps strength, because, although there is a gradual rate of decline, the function of this muscle is preserved for a relatively long time, and may provide large effect sizes to quantify muscle strength loss over time in patients with MND [29, 30]. The quadriceps is, therefore, potentially a sensitive muscle group for objective measurement of disease progression in MND. However, as the quadriceps is one of the strongest muscles of the human body, its assessment is challenging, which leads to high variability among examiners when using the HHD [31].

In this study, we aimed to determine (1) the reliability of PFD as compared to the HHD and (2) the PFD’s ability to reduce possible examiner-induced ceiling effects.

## Methods

### Study population

Study participants consisted of two groups: (1) participants with a diagnosis within the motor neuron disease spectrum (i.e., Amyotrophic Lateral Sclerosis, Progressive Muscular Atrophy or Primary Lateral Sclerosis) [32], and (2) controls without a neurological condition. Participants were excluded from the study if they met one of the following criteria: (1) less than MRC 2 quadriceps strength in both legs, (2) recent or current pain in knee joint or quadriceps muscle, (3) not able to follow test instructions from the examiner, or (4) having another non-MND disorder that affects muscle strength. All patients with MND were recruited from the outpatient clinic of the University Medical Centre Utrecht, The Netherlands. Control participants were recruited from personnel and students from the department of rehabilitation and geriatrics. The study was approved by the Medical Ethics Committee of the UMCU (protocol number 18–243). All study participants gave written informed consent to participate in this study.

### Procedures and measurement techniques

**Fig. 1:**
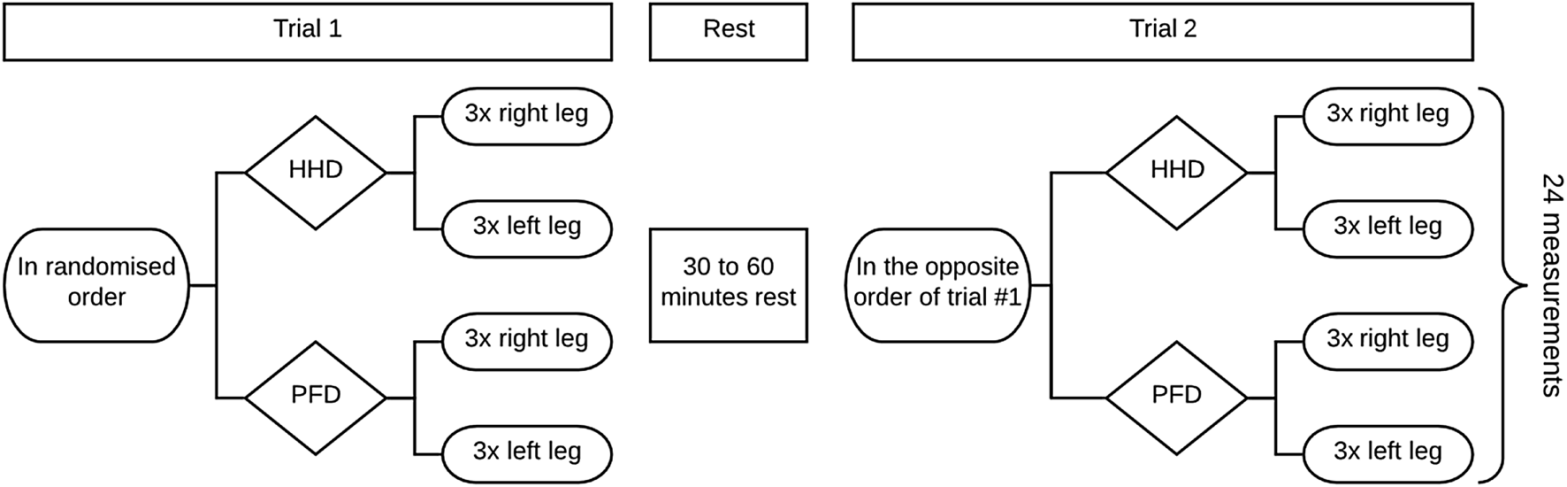
Overview of study procedures

Two examiners (T.G.A. and M.J.H) were certified for isometric HHD muscle testing after (1) completing the ‘Treatment Research Initiative to Cure ALS’ (TRICALS) e-course ‘Isometric muscle testing in ALS’, and (2) satisfactorily completing supervised HHD testing of five control participants. After the registration of patient information and collection of MND-specific characteristics (e.g., ALS functional rating scale [ALSFRS-R] and respiratory functioning), test re-test reliability was assessed in two separate trials on the same day (Fig. 1). Thirty minutes before each trial, participants were requested to refrain from engaging in any strenuous activities. Each trial consisted of six measurements per leg, three with the HHD and three with the PFD. Participants were seated on a chair with back support, hips and knees were kept at 90 degrees. To rule out the influence of arm function, arms were placed in the lap. The starting sequence of the assessment (HHD or PFD) was randomized to minimize the effect of fatigue. All trials within the same participant were conducted by the same examiner, verbally instructing and motivating the patient.

#### Hand-held dynamometry

The HHD (MicroFET 2, HOGGAN Scientific) assessments consisted of three isometric ‘break contractions’ [33], approximately 10 s apart. The HHD was placed 1 centimeter proximal to the midline between the malleoli. If necessary, a towel roll was placed under the knee to prevent the foot from touching the ground. During the maximal contraction, the examiner not only attempted to offer sufficient resistance, but also strived to give a gentile break in the opposite direction of the isometric strength. The score of each measurement, as well as the ability to perform a ‘break’ (classified as break OR unable-to-break), was registered.

#### Portable fixed dynamometer

**Fig. 2:**
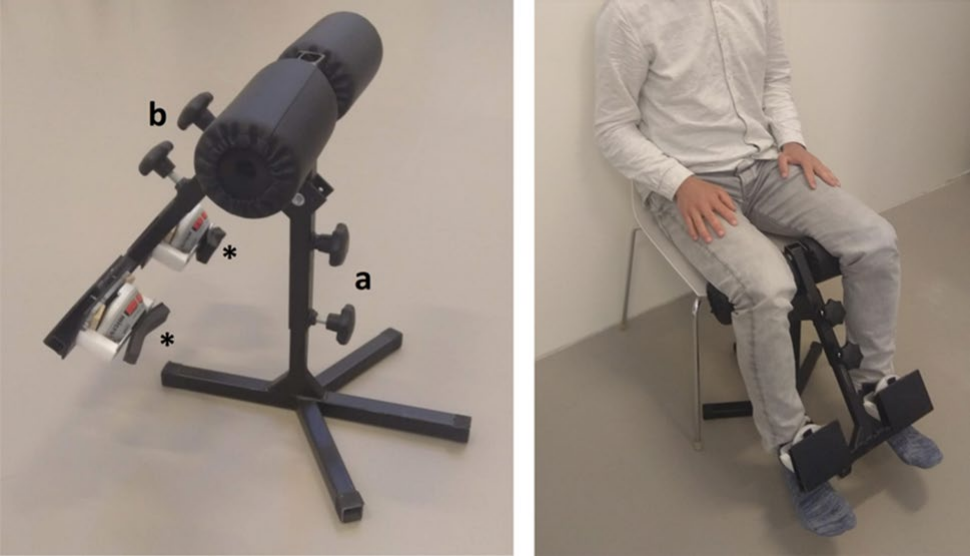
Prototype of the Portable Fixed Dynamometer. A rigid framework with two holders for the MicroFET dynamometers (*) was created to remove the need for an examiner when evaluating quadriceps strength. With the vertical arm (**a**), the height was adjusted to prevent the feet from touching the ground. The diagonal arm (**b**) enabled adjustment of the dynamometer pad to 1 centimeter proximal to the midline between the malleoli

To be able to measure muscle strength of the quadriceps muscle using a portable, but fixed device, we constructed a simple rigid construction with two HHD holders. In Fig. 2 we illustrate the PFD with the HHDs fixed in a rigid, but portable construction. The PFD can be easily placed in front of a (wheel) chair and standardizes the starting position of the knee joint at 90 degrees. The vertical arm of the PFD (indicated by *a,* Fig. 2) can be adjusted to prevent the feet from touching the ground. The arm length of the PFD (indicated by *b*) is adjusted to the length of the participant’s lower leg and positions the HHD. We evaluated muscle strength in a fashion identical to that of the HHD. Importantly, in contrast to the HHD, and due to the nature of the fixed construction, the PFD tests consisted of an isometric ‘make contraction’ as opposed to a ‘brake contraction’ [33].

### Statistical analysis

Data were summarized using the mean with standard deviation (SD) for continuous variables and number with percentage for categorical variables. Mean differences (MD) were calculated between cases and controls using an independent *t* test. For each participant, per trial (i.e., trial 1 or 2) and per method (i.e., HHD or PFD), we selected the highest muscle strength score from the two values which were most similar of the three measurements taken. Test–retest reliability was assessed using (1) the intraclass correlation coefficient (ICC) and its associated standard error of the measurement (SEM), and (2) the Bland–Altman method of agreement [34, 35]. Due to the heteroscedastic nature of the data, we applied a ^10^log-scale transformation, and calculated the mean difference between trials. The ICC was estimated using a linear mixed effects model, incorporating only a fixed intercept and random intercept per subject. The ICC was then calculated as the percentage of the total variation (i.e., sum of the between-subject and within-subject variation) that can be explained by between-subject differences (i.e., the between-subject variation); 95% confidence intervals were obtained by means of bootstrapping (*n* = 1000). The SEM was calculated by taking the square root of the within-subject variation and back-transformed as described elsewhere [35]. Finally, the presence of a ceiling effect was assessed in two steps. First, we assessed the relationship between HHD and PFD measurements with a linear mixed model. This relationship was modelled using a natural spline with four knots. Secondly, we compared the relationship between HHD and PFD measurements with functional loss, using item 8 (walking) of the ALSFRS-R. All analyses were conducted using R. Linear mixed models were fitted and bootstrapped using the *lmer* and *bootMer* functions (R package lme4, version 1.1–21), respectively [36].

## Results

Between 4th April 2018 and 8th March 2019, 88 Dutch participants were enrolled in this study: 45 patients with MND and 43 non-neurological controls; their baseline characteristics are presented in Table 1. The age distribution ranged from 22 to 94 years. In the control subjects, the range of muscle strength was larger for PFD (8 N to 783 N) as compared to HHD (15 N to 414 N). As expected, patients with MND had a significantly lower quadriceps strength compared to the control population (*p* < 0.001). The group difference in quadriceps strength (MND vs controls) was, however, considerably larger on PFD (− 113 N, 95% CI − 52 N to − 174 N, standardized: 0.792) as compared to HHD (− 60 N, 95% CI 23 N to 97 N, standardized: 0.679).

**Table 1:** Baseline characteristics of study population

Characteristic	Patients (*N* = 45)	Controls (*N* = 43)
Age (years)		
Median	62	47
Range	30–84	22–94
Males	27 (60%)	22 (51%)
Body mass index (kg/m^2^)	25 (2)	24 (3)
Muscle strength, average (*N*)		
HHD	216 (94)	275 (80)
PFD	237 (126)	350 (159)
Muscle strength, range (*N*)		
HHD	15–373	128–414
PFD	8–508	91–783
MND subtype		
ALS	41 (91%)	–
PMA	3 (7%)	–
PLS	1 (2%)	–
Bulbar onset	11 (24%)	–
FVC, % predicted—GLI2012	78 (20)	–
Symptom duration (months)		
Median	26	–
Range	8—311	–
Diagnostic delay (months)		
Median	12	–
Range	3—157	–
Riluzole use	37 (82%)	–
ALSFRS-R total score (SD)	35 (7)	–
ΔFRS (points per month)		
Median	0.41	–
Range	0.01–1.94	–

Data are in mean (SD) or no. (%).ΔFRS = 48—ALSFRS-R total score/symptom duration [48]. Prognostic subgroups are based on the ENCALS prediction model [49]

*HHD* hand-held dynamometry, *PFD* portable fixed dynamometry, *MND* motor neuron disease, *ALS* amyotrophic lateral sclerosis, *PMA* progressive muscular atrophy, *PLS* primary lateral sclerosis, *ALSFRS-R* revised ALS functional rating scale

### Reliability HHD and PFD

**Fig. 3:**
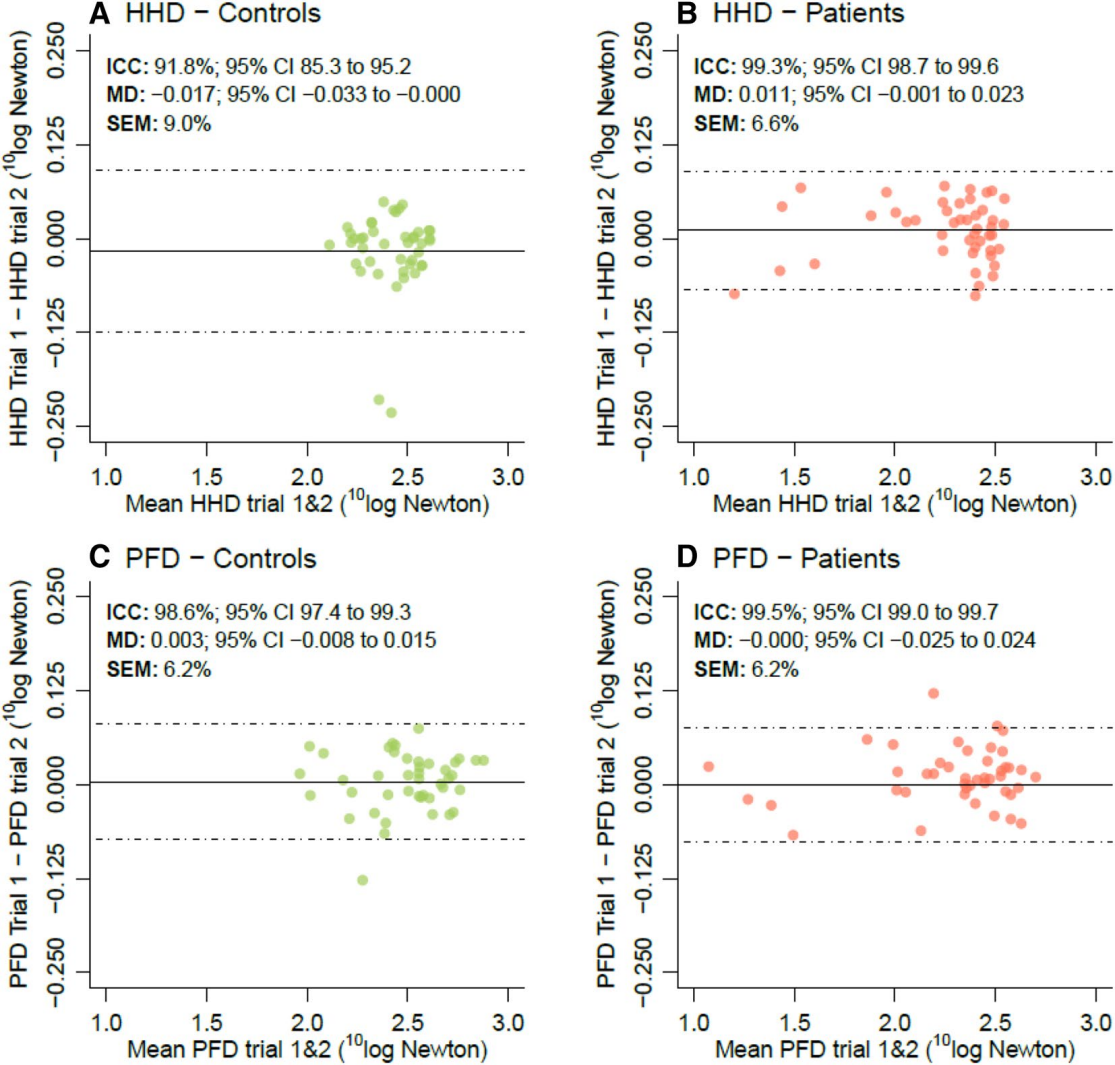
Bland–Altman plots for test–retest reliability. Bland–Altman plots of the HHD (**a–b**) and PFD (**c**–**d**) for non-neurological controls and patients with MND, respectively. Due to the heteroscedastic nature of the data, we applied a ^10^log-transformation. *MD* mean difference between trials 1 and 2, *ICC* intraclass correlation coefficient, *SEM* standard error of measurement, expressed as percentage of the mean on the original scale (Newton) [35]

In Fig. 3, we provide the measurements of reliability. Due to the heteroscedastic nature of the data, we applied a ^10^log transformation on the muscle strength scores. For both the HHD and PFD, no systematic differences were found between trials 1 and 2 as indicated by their mean difference (MD, i.e., mean trial 1—trial 2). The ICC of the PFD was excellent and similar in both patients and controls (ICC _Patients_ 99.5% *vs.* ICC _Controls_ 98.6%). The back-transformed SEM of the PFD was 6.2% of the mean strength in Newton, meaning that test–retest values may vary by as much as ± 12.5% of their mean (i.e., approximately two times the SEM) [35]. Interestingly, the ICC of the HHD was excellent, albeit considerably lower in controls (ICC _Patients_ 99.3% *vs.* ICC _Controls_ 91.8%).

### Examiner-induced ceiling effects in hand-held dynamometry

**Fig. 4:**
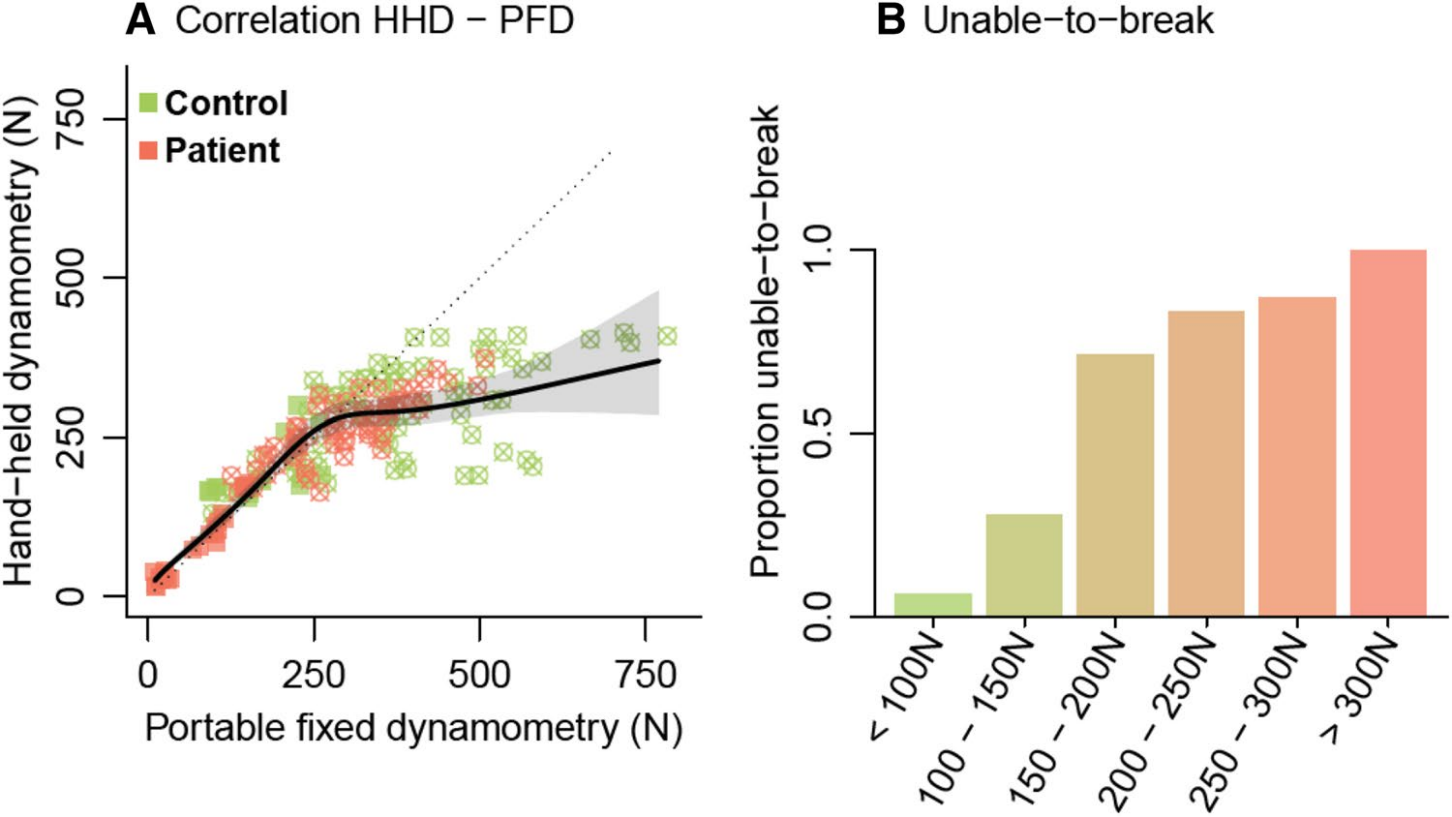
Association between hand-held and portable fixed dynamometry. **a** Association between HHD and PFD measurements. The dotted line represents a correlation of 1 (i.e., HHD measurement = PFD measurement and vice-versa). The black solid line represents the observed association, estimated using a linear mixed effects model. The dots with crosses are data points classified as unable-to-break. **b** Muscle strength based on hand-held dynamometry data was categorized and per sub-group we determined the proportion unable-to-break A clear pattern emerges with the assessor no longer being able to measure the full muscle strength from 300 N upwards (i.e., a proportion of unable-to-break of 1)

Figure 4a shows the relationship between HHD and PFD measurements. For lower values up to 200 N, the PFD and HHD show visually a level of high agreement, resulting in nearly identical strength values. Above 200 to 250 N, the proportion of unable-to-break (Fig. 4b) increases and the correlation between PFD and HHD weakens (as reflected by a deviation from the dashed line). As patients, on average, had lower muscle strength than controls, the correlation coefficient between HHD and PFD was high in patients (Pearson’s *r* 0.94, 95% CI 0.89 to 0.97), whereas in controls it was considerably lower (Pearson’s *r* controls: 0.71, 95% CI 0.52 to 0.83).

**Fig. 5:**
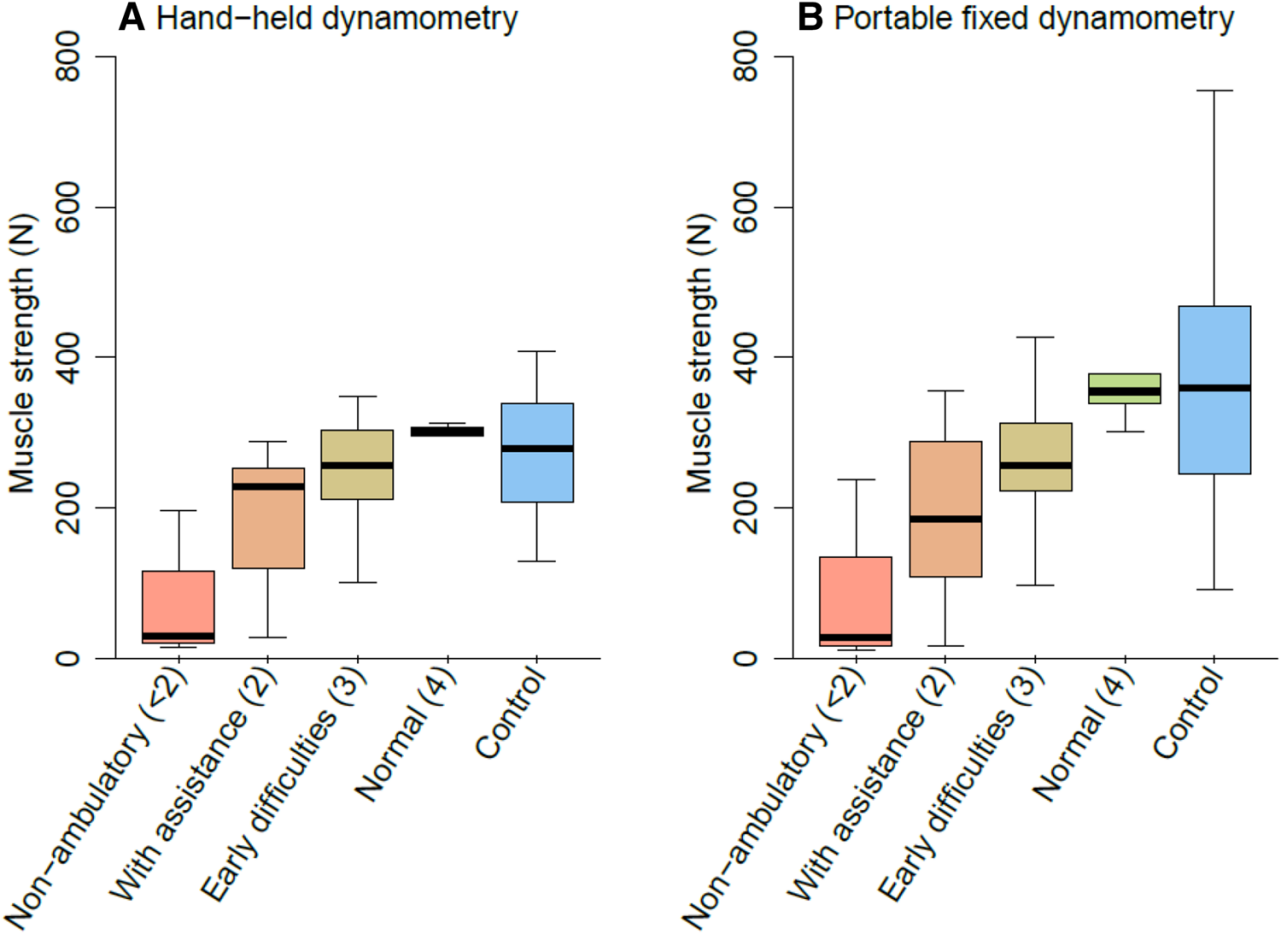
Relationship between functional loss and muscle strength measurements**.** Boxplot of the HHD and PFD measurements in 45 patients with MND and 43 non-neurological controls (blue). Functional loss in MND patients was categorized using self-reported function scores of the ALS functional rating scale item 8 (walking); the score range was 0 to 4, where 0 indicates no leg muscle function (not included) and 4 indicates normal walking function

When HHD and PFD measurements were compared between subgroups based on the score on question 8 (walking) of the ALSFRS-R (Fig. 5), the effect of the ceiling effect became visible. On a group level, the distinction between scores 2, 3 and 4 of item 8 of the ALSFRS-R, did not appear profound for HHD measurements. However, in PFD measurements, muscle strength was approximately linearly related to the reported functional loss.

## Discussion

In this study, we showed that (1) the PFD achieves a high level of precision with an excellent test–retest reliability over a wide range in muscle strength measurements and (2) the PFD has an advantage over hand-held isometric strength measurements as it reduces examiner-induced ceiling effects. Fixating a dynamometer in a simple and portable framework opens the door to standardized self-assessments by patients in their homes, and may eventually decrease the number of hospital visits and reduce the burden to participate in clinical trials.

Optimizing muscle strength testing is important to optimize the evaluation of efficacy of new MND therapies and to contribute to the delivery of remote care [20]. In the field of voluntary muscle strength testing, the Biodex system is considered to be the gold standard [21, 37, 38]. Similar to the Biodex, other systems have been developed that fixate a loading cell or dynamometer in a rigid framework [e.g., Maximum Voluntary Isometric Contraction (MVIC) or Accurate Test of Limb Isometric Strength (ATLIS)]. The MVIC and ATLIS have proven to be reliable in patients with ALS with an excellent intra-rater test variability ranging from 8.6% to 8.9% for the assessment of quadriceps muscle strength.[16, 39] These rigid frameworks are, however, not portable, require visits to the out-patient department, and are still operated by a trained examiner. Other methods that are applied to fixate dynamometers are belt or clamp fixations and stabilization devices [18, 37, 40–44]. Although these have the advantage of being portable and less expensive, unsupervised use has led to inaccurate measurements and patient discomfort [45].

Our results show that the examiner-induced ceiling effect in HHD measurements may be an important source of variability. This is a critical observation as the HHD is a common endpoint in both exploratory and confirmatory clinical trials for ALS [5, 6]. The ceiling effect prevents the investigator from determining the patient’s true strength if the examiner is no longer able to overcome the participant’s muscle strength [10, 15, 21, 22]. This may become particularly problematic in longitudinal settings if patients are assessed by multiple examiners, with each examiner being able to withstand a different amount of force (commonly around 200–300 N) [14, 15]. More importantly, site personnel training and their experience are unlikely to fully eliminate these effects, leading to persistent between-examiner and -site variability. Although the ceiling effects are irrelevant for relatively weak muscle groups, the true strength of major muscle groups like the quadriceps could be significantly underestimated with HHD. Particularly in asymptomatic stages of the disease (as was shown in Fig. 5), this could mean important signals of early disease progression or therapeutic efficacy are missed.

Fixating the HHD in a rigid framework could, therefore, reduce the effect of examiner strength on muscle strength assessments. As is indicated by our results, employing a rigid framework around the HHD increases the range of muscle strength measurements across individuals, especially in early disease. On an individual level, these results are critical as they suggest that the PFD may be able to better track the progression curve of quadriceps strength. On a group-level, this may result in larger effect sizes (e.g., standardized group-differences), which could have significant benefits for clinical trials in terms of sample size. Importantly, despite the increase in range, the test–retest reliability of the PFD remained practically unchanged; implementing the PFD may, therefore, help standardize muscle testing protocols in multicenter settings and reduce site-variability.

Our study does, however, have limitations. As the comparison was limited to PFD *vs.* HHD, it remains to be established how well the PFD performs compared to the gold standard (i.e., Biodex). Previous research indicated a good correlation between HHD and the Biodex for measurements of the quadriceps [21, 46, 47]. Given the strong correlation between HHD and PFD, we expect that the PFD and Biodex will have an equivalent correlation.

The PFD is currently applied to one muscle group and might, therefore, not capture the full extent of motor function loss in patients with MND (e.g., arm weakness). Extensive muscle strength testing is time-consuming and increases patient burden which may lead to significant attrition over time [2, 45]. It is, therefore, critical to minimize the number of assessments, while obtaining sufficient information for clinical decision-making or monitoring disease progression. Dedicated longitudinal studies are required to use a data-driven approach to determine which muscle groups provide complementary information in addition to quadriceps strength monitoring. An important aspect to consider is the current transition to home-based assessments (i.e., remote monitoring); [23–26] portability, cost-effectiveness and the potential for unsupervised, user-friendly assessments should have a prominent place in any future iteration of the PFD.

In conclusion, our study reveals the value of fixed dynamometry in reducing examiner-induced ceiling effects and optimizing the standardization of muscle strength testing to maximize test–retest reliability. The PFD may improve our ability to track disease progression in individual patients and magnify group-level differences. Ultimately, extending the PFD to home-based settings in MND or other neuromuscular diseases, could improve the delivery of remote care, optimize trial efficiency, and reduce the burden to the patient of participating in clinical trials. A prerequisite for independent remote use is further development of the PFD by integrating the load cells and introducing a patient-friendly user interface.

## Data Availability

The corresponding author is able to provide the anonymized data of this study upon reasonable request from qualified investigators.

## Code availability

The corresponding author is able to provide custom R-studio codes that were used for statistical analysis of this study upon reasonable request from qualified investigators.
